# The Treatment of Locomotor Ataxia by Suspension

**Published:** 1889-06

**Authors:** William C. Krauss

**Affiliations:** Attica, N. Y.; 5 Rue Rollin, Paris


### THE TREATMENT OF LOCOMOTOR ATAXIA BY SUSPENSION.

By WILLIAM C. KRAUSS, B. S., M. D., Attica, N. Y.

IN HIS lesson of March 8, 1889, Charcot discussed the results obtained by suspending patients affected with locomotor ataxia. (For the modus operandi, see *Le Progrès Médical*, January 19, 1889, and February 23, 1889. A translation of the latter paper by Dr. Angell appeared in this JOURNAL April, 1889.)

Some five months had then elapsed since this method was introduced in the Salpêtrière, and ninety-one patients were under observation. (This number had increased to 176 April 3, 1889.)

Charcot considered this number, and the length of time, five months, as sufficient to formulate some notions as to the efficacy of this manner of treatment.

The patients who were first subjected to this treatment have shown a gradual and satisfactory improvement in some of the symptoms, but in no case has anything been obtained looking towards a complete recovery. Charcot strongly insists not to consider this mode of treatment curative, but only palliative. By so doing, not only the patient, but also the physician, will be content with the relief obtained.

The symptoms most generally improved are those of the spinal cord; symptoms due to the lesion in the cerebrum and medulla remain unchanged. In the great majority of cases, the lancinating pains of the legs are the first symptoms which yield to this treatment. Generally after ten to fifteen suspensions, the patient finds marked relief. In many cases these pains may disappear altogether. The disappearance of these pains in a case of Pott's disease with tabes undergoing suspension, first suggested to Motchoukowsky the application of the Sayre apparatus to locomotor ataxia. The paresthesia of the feet and legs disappears also quite early. Patients lose the queer sensation of walking on a soft spongy substance, and again feel the firm, hard surface. The girdle pain remains obdurate, as in only one patient was there any relief obtained. Romberg's symptom shows a gradual improvement, although in no case can it be said to have entirely disappeared. Westphal's symptom persists, no change whatever having occurred in the condition of the reflexes. The gait, however, is markedly changed. Patients who formerly came to the Salpêtrière with much assistance, are now able to come alone, unassisted; in some cases coming from a great distance through the crowded city. The vesical and sexual functions reassume, in the majority of cases, their former activity. On the other hand, the ocular symptoms remain unaffected, no improvement or change having been noted. The same applies to the other head symptoms.

It is thus evident that in those cases where the spinal symptoms predominate, and this is the rule, marked improvement may be obtained. Whether this improvement shall be lasting, or only transitory, cannot as yet be said; the patients first to undergo suspension have reported no relapses, no recurrence of former symptoms. While all spinal symptoms are not alike improved, still any relief in some one of the obdurate symptoms of locomotor ataxia is gratefully welcomed by the patient.

As to whether suspension is only an experiment of short duration, or whether it will take a place in the treatment of locomotor ataxia, depends upon how much is expected of it. Considered as a curative agent, it surely will disappoint. Considered as a palliative agent, the patient himself, reflecting upon his condition in former days and that of to-day, will pronounce it the only remedy that gives so much relief in so short a time.

Charcot has also applied suspension in other diseases of the nervous system. In two cases of Friedreich's disease, gratifying results have been obtained. In spastic paraplegias, the first application seemed rather to aggravate the symptoms. Subsequent suspensions have, however, proven beneficial. In Parkinson's disease (paralysis agitans) the patients have experienced much relief. Two cases were under observation, and both are well satisfied with the results of the suspension. The rigidity and stiffness have given way to a great degree. The sensations of *chaleur* (flushings) have abated, the sleep is improved, and the gait has become less fatiguing.

The tremor, however, has shown no sign of disappearing, but remains as persistent as ever.

5 Rue Rollin, Paris.

